# Relationship Between the Xylem Anatomy of Grapevine Rootstocks and Their Susceptibility to *Phaeoacremonium minimum* and *Phaeomoniella chlamydospora*

**DOI:** 10.3389/fpls.2021.726461

**Published:** 2021-10-12

**Authors:** Charis K. Ramsing, David Gramaje, Sara Mocholí, Javier Agustí, Félix Cabello Sáenz de Santa María, Josep Armengol, Mónica Berbegal

**Affiliations:** ^1^Instituto Agroforestal Mediterráneo, Universitat Politècnica de València, Valencia, Spain; ^2^Instituto de Ciencias de la Vid y del Vino (ICVV), Consejo Superior de Investigaciones Científicas, Universidad de la Rioja–Gobierno de La Rioja, Logroño, Spain; ^3^Instituto de Biología Molecular y Celular de Plantas, Consejo Superior de Investigaciones Científicas (CSIC) Universitat Politècnica de València, Valencia, Spain; ^4^Instituto Madrileño de Investigación y Desarrollo Rural, Agrario y Alimentario (IMIDRA), Madrid, Spain

**Keywords:** tolerance, vascular pathogens, Esca, Petri, fungal trunk diseases

## Abstract

Fungal grapevine trunk diseases (GTDs) are some of the most pressing threats to grape production worldwide. While these diseases are associated with several fungal pathogens, *Phaeomoniella chlamydospora* and *Phaeoacremonium minimum* are important contributors to esca and Petri diseases. Recent research has linked grapevine xylem diameter with tolerance to *Pa. chlamydospora* in commercial rootstocks. In this study, we screen over 25 rootstocks for xylem characteristics and tolerance to both *Pa. chlamydospora* and *Pm. minimum*. Tolerance was measured by fungal incidence and DNA concentration (quantified via qPCR), while histological analyses were used to measure xylem characteristics, including xylem vessels diameter, density, and the proportion of the stem surface area covered by xylem vessels. Rootstocks were grouped into different classes based on xylem characteristics to assess the potential association between vasculature traits and pathogen tolerance. Our results revealed significant differences in all the analyzed xylem traits, and also in DNA concentration for both pathogens among the tested rootstocks. They corroborate the link between xylem vessels diameter and tolerance to *Pa. chlamydospora*. In *Pm. minimum*, the rootstocks with the widest xylem diameter proved the most susceptible. This relationship between vasculature development and pathogen tolerance has the potential to inform both cultivar choice and future rootstock breeding to reduce the detrimental impact of GTDs worldwide.

## Introduction

Esca and Petri diseases are two major fungal grapevine trunk diseases (GTDs) that currently and significantly threaten grapevine production (Gramaje et al., 2018). These diseases are present in every grape-growing region worldwide. The complex etiology of such diseases comprises many fungal species. These include the pathogen *Phaemoniella chlamydospora* and several *Phaeoacremonium* species with *Phaeoacremonium minimum* being the most widely distributed and the most commonly isolated species of this genus affecting grapevine (Bertsch et al., 2013; Gramaje et al., 2015, 2018).

Esca symptoms can emerge in either mild or severe forms. Severe esca is also called “apoplexy” and can involve abrupt wilting and even grapevine death. Milder or chronic forms usually feature deteriorating foliage. This begins with interveinal chlorosis that progresses to necrotic tissue and results in “tiger-striped” leaves. Berries may display scattered spots known as “black measles.” The internal wood symptoms of affected plants include white soft-rot surrounded by a dark line and cinnamon to black spots (Bertsch et al., 2013; Gramaje et al., 2018).

Petri disease more frequently affects grafted vines in grapevine nurseries and young vines in newly planted vineyards and causes plant stunting and dieback (Gramaje and Armengol, 2011; Gramaje et al., 2018). Additional external symptoms include delayed bud break, weak vegetative growth, and occasional interveinal chlorosis that leads to necrosis and leaf wilt. Internal symptoms are often characterized by dark discoloration on the grapevine trunk and cordons, and by brown to black vascular streaking (Mostert et al., 2006b; Gramaje et al., 2010, 2018).

Esca and Petri diseases have become increasingly prevalent in recent years due to several factors. Specifically, (i) the worldwide grapevine planting boom in the 1990s, (ii) changes in cultural practices that favor fungal infections, and (iii) lack of effective chemicals preventing fungal infections in both nurseries and vineyards (Gramaje et al., 2018; Mondello et al., 2018). Unfortunately, there are no current viable or universal methods to manage these diseases, thus contributing to their widespread across all vine-growing areas (Mondello et al., 2018).

Host resistance is almost always the ideal management method for plant pathogens, especially in perennial crops like grapevine, where re-establishing fields year after year makes production costly (Gramaje et al., 2018). The estimated worldwide annual financial cost of replacing plants that have died of GTDs is more than € 1,132 billion (US$ 1.502 billion) (Hofstetter et al., 2012). However, while the susceptibility of rootstocks and cultivars differ to some GTDs, no cultivar or species in *Vitis* has been found to express complete resistance (Eskalen et al., 2001; Feliciano et al., 2004; Gramaje et al., 2010; Martínez-Diz et al., 2019). Instead, all grapevine rootstocks and cultivars can be potentially infected by GTD fungi, but symptom expression and severity vary among cultivars. Quantitative resistance does not prevent infection, but limits crop losses and damage and reduce the epidemic over time (Suthman et al., 2007; Poland et al., 2008; St. Clair, 2010). Quantitative resistance is typically not strain-specific and thus, tends to be effective across all the strains of a pathogen population (Niks et al., 2015).

A better understanding of disease development is essential to identify novel genetic resources for resistance against Esca and Petri diseases. Screening studies to evaluate rootstock susceptibility to *Pa. chlamydospora* and *Pm. minimum* have been conducted by artificial inoculation under field (Gramaje et al., 2010), under controlled conditions using *in vitro* grapevine shoots (Zanzotto et al., 2008), and greenhouse cuttings (Eskalen et al., 2001). Although these studies were useful, they yielded inconsistent results and did not offer any information about pathogen colonization or rootstock characteristics that might be associated with different susceptibility levels. Identifying physiological characteristics linked with tolerant rootstocks would greatly help breeding toward disease tolerance (Nelson et al., 2018).

The physiological and histochemical alterations associated with *Pa. chlamydospora* infections are subjects of more recent interest. Studies employing green fluorescent protein (GFP)-transformed *Pa. chlamydospora* and *Pm. minimum* have demonstrated that the fungi are found mainly in woody tissue and concentrates around occluded xylem vessels (Landi et al., 2012; Pierron et al., 2015). *Pa. chlamydospora* is known to induce the host plant to produce tyloses containing various tannins and phenolic compounds, which may explain these obstructions (Lorena et al., 2001). Similar occlusion structures are found in many fungal wilt diseases and are considered to plant defense mechanisms that seal off and trap pathogens in an enclosed space where defense compounds accumulate (Fradin and Thomma, 2006). These findings indicate that the plant vasculature may play a more relevant role in disease development than expected. Indeed, one key determinant of plant resistance to vascular infections lies in the ability of the host to successfully compartmentalize invaders at the xylem level (Pouzoulet et al., 2020a). More specifically, the impact of xylem vessel diameter on compartmentalization efficiency and, thus, on vascular pathogen movement has been analyzed for *Pa. chlamydospora* in grapevine (Pouzoulet et al., 2017, 2020a). Recent studies into grapevine xylem anatomy have shown that grapevine rootstocks display varying xylem vessel diameters, and the density of xylem vessels with diameters above 120 μm correlates with the *Pa. chlamydospora* DNA concentration. These observations support the hypothesis that a wider xylem vessel would be harder to obstruct (Pouzoulet et al., 2014, 2017). Comparable results have also been found in Dutch elm disease caused by the vascular pathogen *Ophiostoma novo-ulmi* (Solla and Gil, 2002; Venturas et al., 2014).

There is a need to provide further insights into the idea that vasculature anatomy is a driver in grapevine tolerance to esca and Petri diseases. In our study, shoots from grapevine rootstocks representing different agronomical characteristics and *Vitis* spp. crosses were inoculated with *Pa. chlamydospora* and *Pm. minimum* to determine their susceptibility to these pathogens using fungal isolation and DNA concentration (the latter determined by qPCR) as the main criteria. Xylem anatomy parameters such as xylem density, xylem vessels surface area, and vessel diameter were also measured. The specific objectives of this study were to (i) evaluate differences in pathogen tolerance to *Pa. chlamydospora* and *Pm. minimum* and in xylem vessel characteristics among the studied rootstocks, and (ii) investigate the relationship between tolerance to fungal pathogens and xylem anatomy.

## Materials and Methods

### Grapevine Rootstock Inoculation

Shoots from different grapevine rootstocks were selected to represent different agronomical characteristics and *Vitis* spp. crosses (Table 1). Referenced well-characterized isolates of *Pa. chlamydospora* (Pch-184, obtained in 2004 at Sinarcas, Valencia, from Tempranillo variety grafted onto 110 R rootstock) and *Pm. minimum* (Pal-45, obtained in 2002 at Argamasilla de Alba, Ciudad Real, from Tempranillo variety grafted onto Richter 110 rootstocks) were used for inoculation. These isolates were maintained in 15% glycerol solution at −80°C into 1.5 ml cryovials at the fungal collection of the Instituto Agroforestal Mediterráneo—Universitat Politècnica de València (IAM-UPV) (Spain).

**Table 1:** List of the rootstocks used in the study.

**Rootstock**	**Parents**	**Origin^a^**	***Phaeomoniella chlamydospora* inoculation^b^**	***Phaeomoniella chlamydospora* qPCR**	***Phaeoacremonium minimum* inoculation**	***Phaeoacremonium minimum* qPCR**	**Histological analysis**
Berlandieri resseguier 1	*V. berlandieri*	El Encín	*	*	*	*	*
Blanchard 1	*V. berlandieri* × *V. vinifera*	El Encín	*	*	*	*	*
Cardeden 31		El Encín	*		*		
Castel 14539	(*V. vinifera* Chasselas × *V. rupestris*) × *V. vinifera* Chasselas	El Encín	*		*		
Castel 196–17	(*V. vinifera* × *V. rupestris*) × *V. riparia*	El Encín	*	*	*	*	*
Castel 6736	*V. riparia* × *V. rupestris*	El Encín	*	*	*	*	*
Castel 6971	*V. riparia* × *V. rupestris*	El Encín	*	*	*	*	*
Castel 7605	*V. riparia* × *V. berlandieri*	El Encín	*	*	*	*	*
Couderc 1202	*V. vinifera* × *V. rupestris*	El Encín	*	*	*	*	*
Couderc 161–49	*V. riparia* × *V. berlandieri*	Viveros Villanueva	*	*			*
COUDERC 1616	*V. longii* × *V. riparia*	El Encín	*		*	*	*
Couderc 3309	*V. riparia* × *V. rupestris*	El Encín	*		*		
Couderc 404	*V. vinifera* × *V. rupestris*	El Encín	*		*		
Couderc 601	Bourrisqouou × *V. rupestris*	El Encín	*		*		
Couderc 9	(*V. riparia* × *V. rupestris*) × *V. vinifera*	El Encín	*		*		
Escuela montpellier 333	*V. vinifera* × *V. berlandieri*	El Encín	*	*	*	*	*
Evex jerez 13–5	*V. berlandieri*	El Encín	*	*	*	*	*
Fercal	*V. berlandieri* × (*V. berlandieri × * Novo Mexicana)	El Encín	*	*	*	*	*
Foex 34-E	*V. berlandieri* × *V. riparia*	El Encín	*		*		
Grezot G1	(*V. longii* × *V. riparia*) × *V. rupestris*	El Encín	*	*	*	*	*
Grimaldi 791	*V. vinifera* × (*V. riparia* × *V. rupestris*)	El Encín	*		*	*	*
Malague 44–53		El Encín	*		*		
Martinez zaporta 5A	*V. vinifera* Chasselas × *V. berlandieri*	El Encín	*		*		
Millardet grasset 41B	*V. vinifera* × *V. berlandieri*	El Encín	*	*	*	*	*
Millardet 145	*V. vinifera* × (Cordifolia-Rupestris Grasset)	El Encín	*		*		
Millardet 33A-1	*V. vinifera* × *V. rupestris*	El Encín	*		*	*	*
MILLARDET 453	Aramon × Millardet	El Encín	*		*		
Millardet grasset 19–62	*V. vinifera* × *V. berlandieri*	El Encín	*		*		
Millardet grasset 420A	*V. berlandieri* × *V. riparia*	El Encín	*	*	*		*
Oberlin 595	*V. riparia* × *V. vinifera*	El Encín	*		*		
Paulsen 1103	*V. berlandieri* × *V. rupestris*	El Encín	*		*		
Paulsen 1447	*V. berlandieri* × *V. rupestris*	El Encín	*		*		
Ramsey		INRA Montpellier	*				
RG1		Vitis Navarra	*		*		
RG10		Vitis Navarra	*		*		
RG2		Vitis Navarra	*		*		
RG3		Vitis Navarra	*		*		
RG4		Vitis Navarra	*		*		*
RG6		Vitis Navarra	*		*		
RG7		Vitis Navarra	*		*		
RG8		Vitis Navarra	*		*		
RG9		Vitis Navarra	*		*	*	*
Richter 99	*V. berlandieri* × *V. rupestris*	El Encín	*		*		
Richter 110	*V. berlandieri* × *V. rupestris*	El Encín	*	*			*
Richter 31	*V. berlandieri* × Novo Mexicana	El Encín	*	*	*	*	*
Riparia grand glabre	*V. riparia*	El Encín	*	*	*	*	*
Ruggeri 131	*V. berlandieri* × *V. rupestris*	El Encín	*		*		
Ruggeri 140	*V. berlandieri* × *V. rupestris*	El Encín	*	*	*	*	*
Ruggeri 267	*V. berlandieri* × *V. riparia*	El Encín	*	*	*	*	*
Ruggeri 343	*V. berlandieri* × *V. riparia*	El Encín	*	*	*	*	*
Rupestris du lot	*V. rupestris*	El Encín	*		*		
Rupestris fort worth 1	*V. rupestris*	El Encín	*	*	*	*	*
Seibel 397	Herbemot touzan × Sauvignon	El Encín	*		*		
SO4	*V. berlandieri* × *V. riparia*	El Encín	*	*	*	*	*
Teleki 10A	*V. berlandieri* × *V. rupestris*	El Encín	*		*		
Teleki-Kober 5BB	*V. berlandieri* × *V. riparia*	El Encín	*	*			*

a *El Encín, Alcalá de Henares (Madrid, Spain); Viveros Villanueva, Larraga (Navarra, Spain); Vitis Navarra, Larraga (Navarra, Spain)*.

b Analysis performed = ^*^.

Rootstock shoots were cut into smaller cutting fragments (10 cm length) with at least one terminal node. The base of these fragments was immersed for 30 min in *Pa. chlamydospora* and *Pm. minimum* spore suspensions (10^6^ conidia/ml) obtained from the above-mentioned isolates based on the methodology described by Gramaje et al. (2010). Nine cuttings per cultivar and fungal species were inoculated and nine cuttings were immersed in sterile distilled water as negative controls.

The inoculated cuttings were randomly distributed in a hydroponic culture system as described by Sosnowski et al. (2016). These cuttings were placed inside holes made in 2 cm-thick polystyrene boards to ensure that the base of cuttings dropped ~1 cm below the boards. These boards were floated on water dosed with a soluble fertilizer (25% Hoagland solution) in plastic tubs inside a plant growth chamber. The liquid substrate received a continuous oxygen supply by an aquarium air pump. Cuttings were maintained at ~25°C for 45 days. The experiment was repeated once. The extent of fungal colonization on shoots was evaluated 45 days after inoculation by pathogen isolation and quantifying the amount of fungal DNA by a qPCR assay.

### Fungal Isolation

Cuttings were collected 45 days after inoculation. The bark was removed using a sharp knife and the exposed wood was surface disinfected for 1 min in 1.5% sodium hypochlorite solution before being rinsed twice in sterile distilled water. For each cutting, wood discs (4 mm) were cut using sterile secateurs at a distance of 4 cm above the inoculated base to assess the presence of *Pa. chlamydospora* or *Pm. minimum*. One-half of each disc was used for pathogen isolation and the other half for DNA extraction (Table 1).

For fungal isolation purposes, six small segments were plated on potato dextrose agar (PDA; Biokar-Diagnostics, Zac de Ther, France) supplemented with 0.5 g/L of streptomycin sulfate (Sigma-Aldrich, St. Louis, MO, USA) (PDAS). Plates were incubated at 25°C in the dark for 7 to 10 days, and all the colonies were transferred to PDA. A cutting was considered infected when at least one positive isolation point was obtained for the inoculated pathogens. Identification of *Pa. chlamydospora* or *Pm. minimum* was based on the colony morphology (Crous and Gams, 2000; Mostert et al., 2006a). By considering these results, the disease incidence percentage was estimated as the number of infected cuttings of all the total inoculated. Of all the non-inoculated controls, one additional wood disk from each rootstock cutting was taken and kept for further histological analyses.

### DNA Extraction and Quantification

Bark and pith were removed from wood sections with a sterile scalpel before genomic DNA (gDNA) was obtained. A total initial wood mass of 50 mg was analyzed. Each sample was homogenized using a mortar and pestle and placed in 2 ml tubes containing 2 and 4, 3- and 2.35-mm-diameter, respectively, tungsten carbide beads (Qiagen, Hilden, Germany), and 500 μl of cetyl trimethylammonium bromide (CTAB) extraction buffer [2% CTAB, 100 mM Tris-HCl, pH 8.0, 20 mM ethylenediaminetetraacetic acid (EDTA), 1.4 M NaCl, and 1% polyvinylpyrrolidone (PVP)]. Tubes were placed in a FastPrep^®^ (MP Biomedicals, Santa Ana, CA, USA) at 124 Hz for 30 s. Subsequently, the DNA extraction procedure was conducted as described by Saito et al. (2013).

For pathogen DNA quantification, the total DNA of the inoculated isolates grown in PDA was extracted with the EZNA Plant Miniprep kit (Omega Bio-Tek, Norcross, GA, USA). Before DNA extraction, the sample was homogenized in 2 ml tubes following the same procedure described before, but with 600 μl of P1 buffer (provided in the kit) instead of CTAB buffer. The concentration (ng/μl) of the obtained gDNA was quantified with the Qubit Fluorometric Quantitation kit (Life Technologies, Carlsbad, CA, USA), which resulted in 18.2 and 5.5 ng/μl for *Pa. chlamydospora* and *Pm. minimum*, respectively. Seven 1:10-fold serial dilutions of gDNA were prepared and used as standards.

Quantitative polymerase chain reaction assays were performed in a final 25 μl volume, and the reaction mixtures contained 12.5 μl of TB Green™ Premix Ex Taq™ (2x) (Tli RNaseH Plus; Takara Bio Inc., Shiga, Japan) and 2 μl of template DNA. The primer sets PchQF/R and PalQF/R described by Pouzoulet et al. (2013) to detect *Pa. chlamydospora* and *Pm. minimum*, respectively, were used at a final concentration of 0.5 μM. Experiments were conducted in a Rotor-Gene Q 5plex HRM instrument (Qiagen), and the reaction conditions were initial denaturation at 95°C for 1 min, followed by 40 cycles of 95°C for 15 s, and 62°C for 45 s. Additional melting analysis from 50 to 99°C was performed to confirm correct product amplification.

The quantification cycle (Cq) value for each standard gDNA sample was calculated by the Rotor-Gene Q Series software (version 2.3.1) to generate a standard curve for the quantification of *Pa. chlamydospora* and *Pm. minimum*, and to estimate the limit of detection. Both standards and samples were analyzed using four technical replicates and the nomenclature for interpreting all the qPCR results followed the MIQE guidelines, as described by Bustin et al. (2009).

### Histological Analysis

For a selected number of rootstocks (Table 1), four 3–5 mm-stem fragments were collected and vacuum-infiltrated by fixation for 15 min in 70% EtOH. Samples were then processed for dehydration, eosin staining, and paraffin infiltration using a Leica TP1020 tissue processor (Leica Biosystems, Buffalo Grove, IL, USA). Subsequently, paraffin blocks containing the eosin-stained samples were made and sectioned using a Leica microtom (Leica Biosystems). In all cases, 60 μm sections were produced, floated in 40°C water, and mounted on slides, which were left overnight on a 40°C heated plate. Samples were then de-parafinned through two histoclear baths. Histoclear was removed with 100% ethanol and coverslips were placed on samples and fixed with Merck-Glass fixing media (Merck, Kenilworth, NJ, USA). For the quantitative analyses, photographs were taken for each sample using a Leica DM5000 microscope (Leica Biosystems). For each sample, the diameter and area of at least 100 vessels were measured in both lateral and dorsal interfascicular regions using the Fiji free software. Xylem vessel area occupancy, namely, the xylem vessel surface, was calculated for each analyzed interfascicular region as the total vessel area/total interfascicular region area ratio. The mean xylem vessel diameter (μm), xylem vessel density (no. of vessels/mm^2^), and xylem vessel surface (mm^2^ xylem/total mm^2^ surface area) values were obtained for each rootstock.

### Data Analysis

Statistical analyses were run within each variable, both independent and dependent, to test if there were significant differences between rootstocks. Data distribution was right-skewed and justified by running a Kruskal-Wallis test for all the variables except xylem density. Dunn's and Wilcoxon rank sum tests were run for the *post-hoc* analyses. Xylem density was assessed by a one-way ANOVA followed by Tukey's *post-hoc* HSD test.

The imbalance between the number of replicates per sample used for the independent and dependent variables made a direct comparison between both variables difficult. Whereas four replicates per rootstock were used for the histological data (independent variables), from four to sixteen replicates per rootstock were employed for the DNA concentration (dependent variable). To mitigate this issue, the independent variables were sorted into classes to convert them from quantitative variables into qualitative factors based on Pouzoulet et al. (2017). Five classes were established for each variable (diameter, density, surface) and assigned as follows: five diameter classes (<44, 45–54, 55–64, 65–74, ≥75 μm), five density classes [<40, 40–49, 50–59, 60–69, ≥70 (no. vessels/mm^2^)], and five surface classes [0.10–0.14, 0.15–0.19, 0.20–0.24, 0.25–0.30, ≥0.31 (mm^2^ xylem/total mm^2^)]. Kruskal-Wallis tests were run for the histological variable DNA concentration combination, followed by Dunn's and the Wilcoxon ranked sum *post-hoc* tests. To see if there were any differences in incidence and DNA concentration and if histological factors were common between crosses, rootstock varieties were sorted into their parent crosses and the same statistical tests were run. The Kendall rank correlation test was used to investigate the potential correlation between histological traits.

All the data analyses were run in RStudio 1.3.959 with R 4.0.0 (R Core Team, 2020). Essential packages included “agricolae,” “ARTool,” “dplyr,” “FSA,” “ggplot2,” “ggpubr,” and “grDevices” (Wickham, 2016; de Mendiburu, 2019; Kassambara, 2020; Kay and Wobbrock, 2020; Wickham et al., 2020; Ogle et al., 2021).

## Results

### Pathogen Tolerance

No significant rootstock effect was observed on either *Pa. chlamydospora* or *Pm. minimum* incidence based on the pathogen isolation from the inoculated cuttings (*P* = 0.1451 and *P* = 0.4739, respectively). All the rootstocks were infected, except Castel 6971 cuttings inoculated with *Pm. minimum*, which had no positive point of isolation. Incidence results vastly varied, with percentage value ranges of 5.5–86.7 and 0–88.9% for *Pa. chlamydospora* and *Pm. minimum*, respectively. Very low *Pa. chlamydospora* incidence levels (<17%) were observed for rootstocks Cardeden 31, Castel 7605, Couderc 601, Paulsen 1447, and Richter 99. Low *Pm. minimum* incidence levels (<17%) were obtained for rootstocks Couderc (9, 1606, and 3309) and Teleki 10A. Incidence levels below 17% were found for Couderc 404, Grimaldi 791, RG10, and Richter 31 rootstock cuttings inoculated with both pathogens (Supplementary Figure 1).

**Figure 1:**
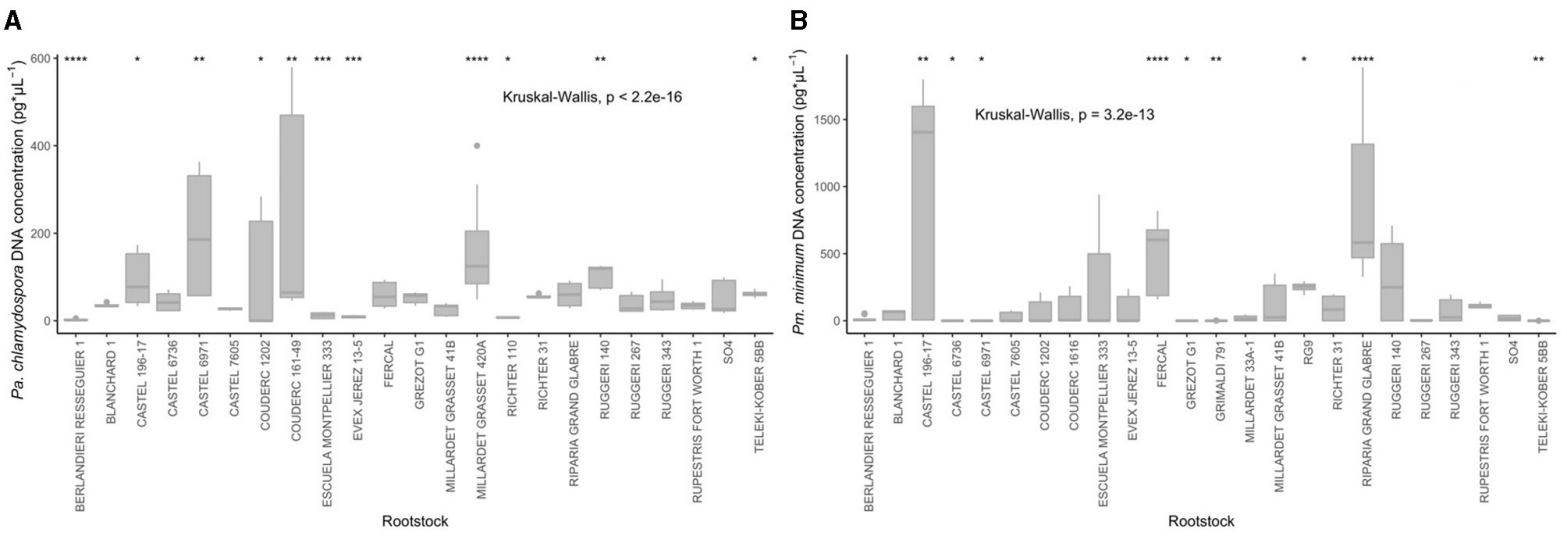
Box plots of the fungal DNA concentration found in differing grape rootstock varieties. **(A)** *Phaeomoniella chlamydospora* DNA concentration in pg/μl considering an initial wood mass for the analysis of 50mg. **(B)** *Phaeoacremonium minimum* DNA concentration in pg/μl considering an initial wood mass for the analysis of 50mg. Significance (*P < 0.05, **P < 0.01, ***P < 0.001, and ****P < 0.0001) determined by the Wilcoxon rank sum test.

The analysis showed significant differences between rootstocks for both pathogens' DNA concentration detected in the inoculated cuttings (*P* < 0.0001, Figure 1). The average DNA concentration values ranged from 2.4 to 163.2 pg/μl and from 0.04 to 916.5 pg/μl for *Pa. chlamydospora* and *Pm. minimum*, respectively. Significantly lower *Pa. chlamydospora* DNA concentrations (<15 pg/μl) were detected in rootstocks Berlandieri Resseguier 1, Escuela Montpellier 333, Evex Jerez 13-5, and Richter 110 (*P* < 0.0001, *P* < 0.001, *P* < 0.001, and *P* < 0.05, respectively) while significantly higher *Pa. chlamydospora* DNA concentrations (>160 pg/μl) were detected in Millardet Grasset 420A, Couderc 161–49, and Castel 6971 (*P* < 0.0001, *P* < 0.001, *P* < 0.001, respectively) (Figure 1A, Supplementary Table 1). For *Pm. minimum*, significantly lower DNA concentrations (<1 pg/μl, *P* < 0.05) were detected in rootstocks Castel 6736 and 6971, Grezot G1, Grimaldi 791, and Teleki Kober 5BB, while significantly higher concentrations (<400 pg/μl) were recorded in rootstocks Castel 196-17, Fercal, and Riparia Grand Glabre (*P* < 0.01, *P* < 0.0001, and *P* < 0.0001, respectively) (Figure 1B, Supplementary Table 1).

### Xylem Anatomy

**Figure 2:**
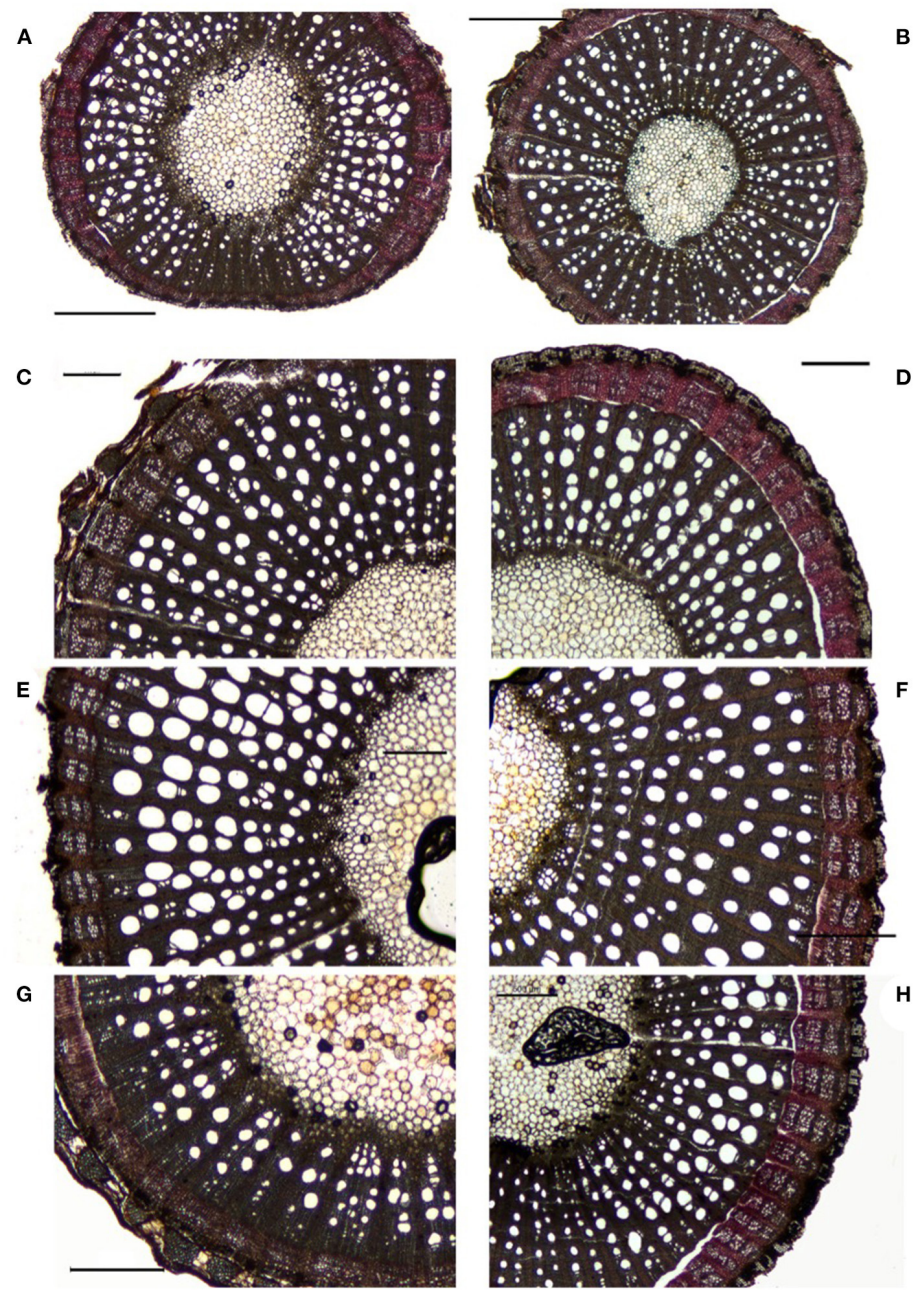
Image analysis for the different rootstocks 60 μm sections. Blanchard 1 **(A)**, Castel 6736 **(B)**, Grezot G1 **(C)**, Rupestris Fort Worth 1 **(D)**, RG9 **(E)**, Richter 110 **(F)**, Grimaldi 791 **(G)**, and SO4 **(H)**. Horizontal bars = 500μm.

**Figure 3:**
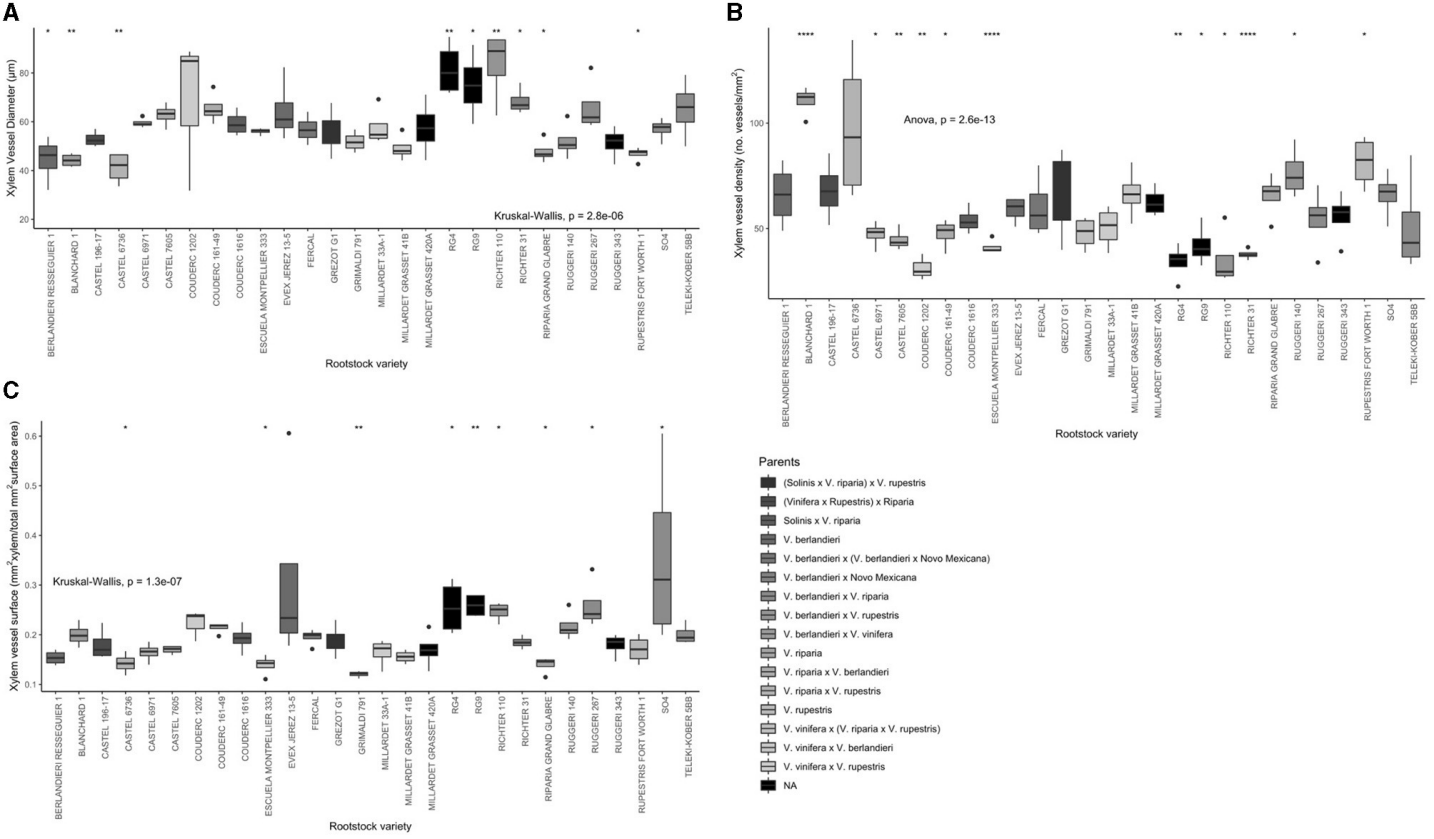
Box plots of the histological traits for each rootstock variety. **(A)** Xylem vessel diameter (μm). **(B)** Xylem vessel density (number of vessels/mm^2^ xylem). **(C)** Proportion of stem surface area covered by xylem vessels (mm^2^ xylem vessels/total mm^2^ vascular surface area). Significance (*P < 0.05, **P < 0.01, and ****P < 0.0001) determined by the Wilcoxon rank sum test for **(A,C)** and unpaired *T*-test for **(B)** (*P* < 0.05). Gray-scale color coded by rootstock parent cross (Solinis = *V. longii*).

Apart from the visual differences among xylem vessel characteristics (Figure 2), a significant rootstock effect was observed for all the xylem anatomy parameters, including vessels diameter, xylem vessel density, and surface area (*P* < 0.01). The average xylem vessel diameter values ranged from 41.1 to 83.6 μm, with RG4, RG9, and Richter 110 showing significantly wider xylem vessels (<75 μm, *P* < 0.01, *P* < 0.05, and *P* < 0.01 respectively) (Figure 3A, Supplementary Table 2). The average xylem vessel density values ranged from 31.1 to 110.5 vessels/mm^2^ with Blanchard 1 having significantly dense vessels (110.5 vessels/mm^2^, *P* < 0.0001; Figure 3B, Supplementary Table 2). The average xylem vessel surface values ranged from 0.1 to 0.4 mm^2^ xylem/total mm^2^ surface area with RG4, RG9, Richter 110, Ruggeri 267, and SO4 obtaining significantly higher surface values (<0.245 mm^2^ xylem/total mm^2^ surface area, *P* < 0.05; Figure 3C, Supplementary Table 2). Interestingly, rootstocks RG4, RG9, and Richter 110 had significantly different values for each histological parameter and significantly wider vessel diameters (<75 μm, *P* < 0.05), significantly lower xylem densities (<41 vessels/mm^2^, *P* < 0.05) and significantly high xylem surface values (<0.245 mm^2^ xylem/total mm^2^ surface area, *P* < 0.05; Figures 3A–C, Supplementary Table 2).

**Figure 4:**
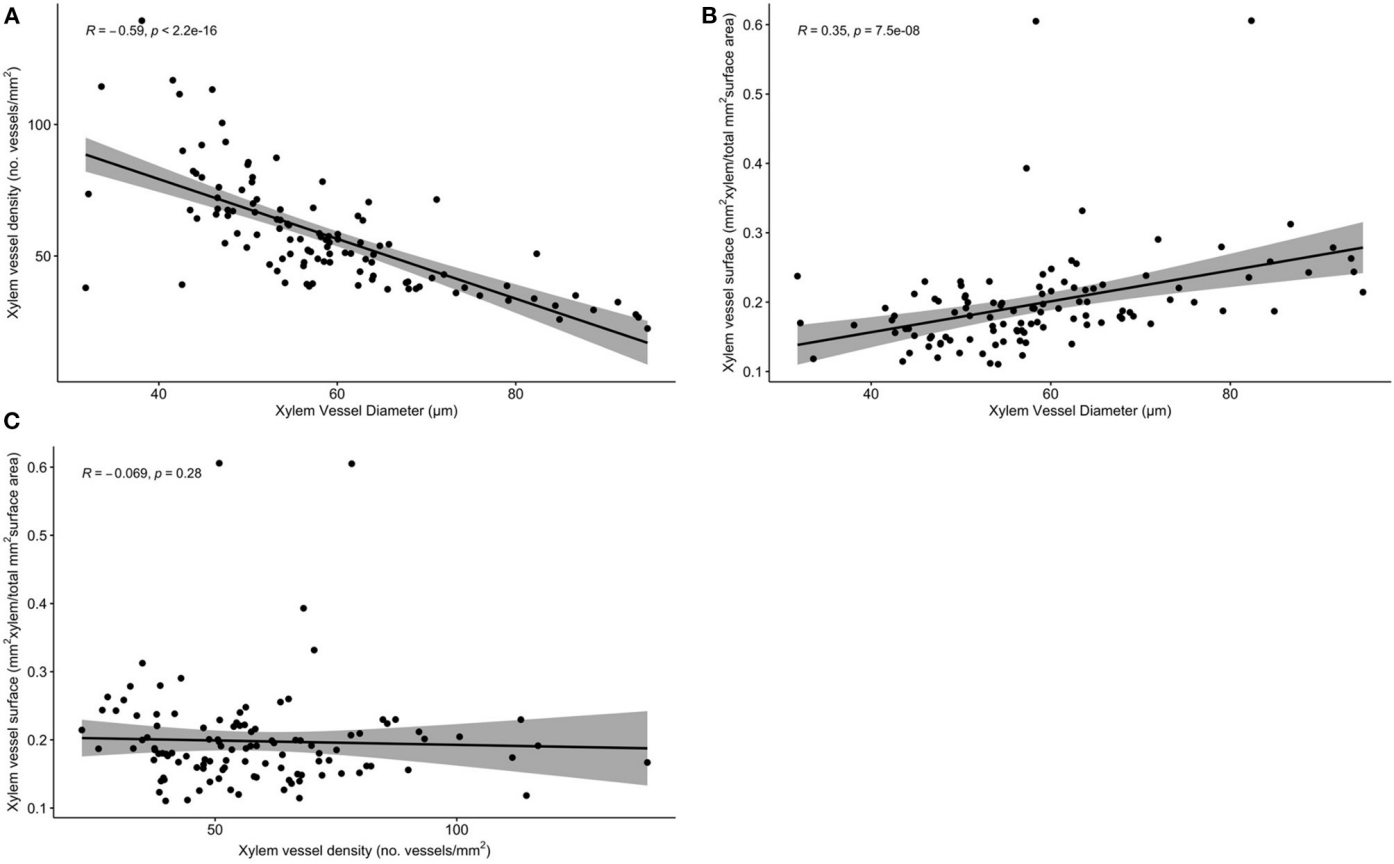
Correlation plots between histological traits using the Kendall rank correlation coefficient. **(A)** Xylem vessel diameter correlated with xylem vessel density. **(B)** Xylem vessel diameter correlated with xylem vessel surface area. **(C)** Xylem vessel density correlated with xylem vessel surface area.

A significant correlation was found between vessels diameter and xylem density (*R* = −0.59, *P* < 0.01) and between vessel diameter and xylem surface (*R* = 0.35, *P* < 0.01; Figures 4A,C). In contrast, no significant correlation was observed between xylem density and surface (R = −0.069, *P* = 0.28; Figure 4B).

**Figure 5:**
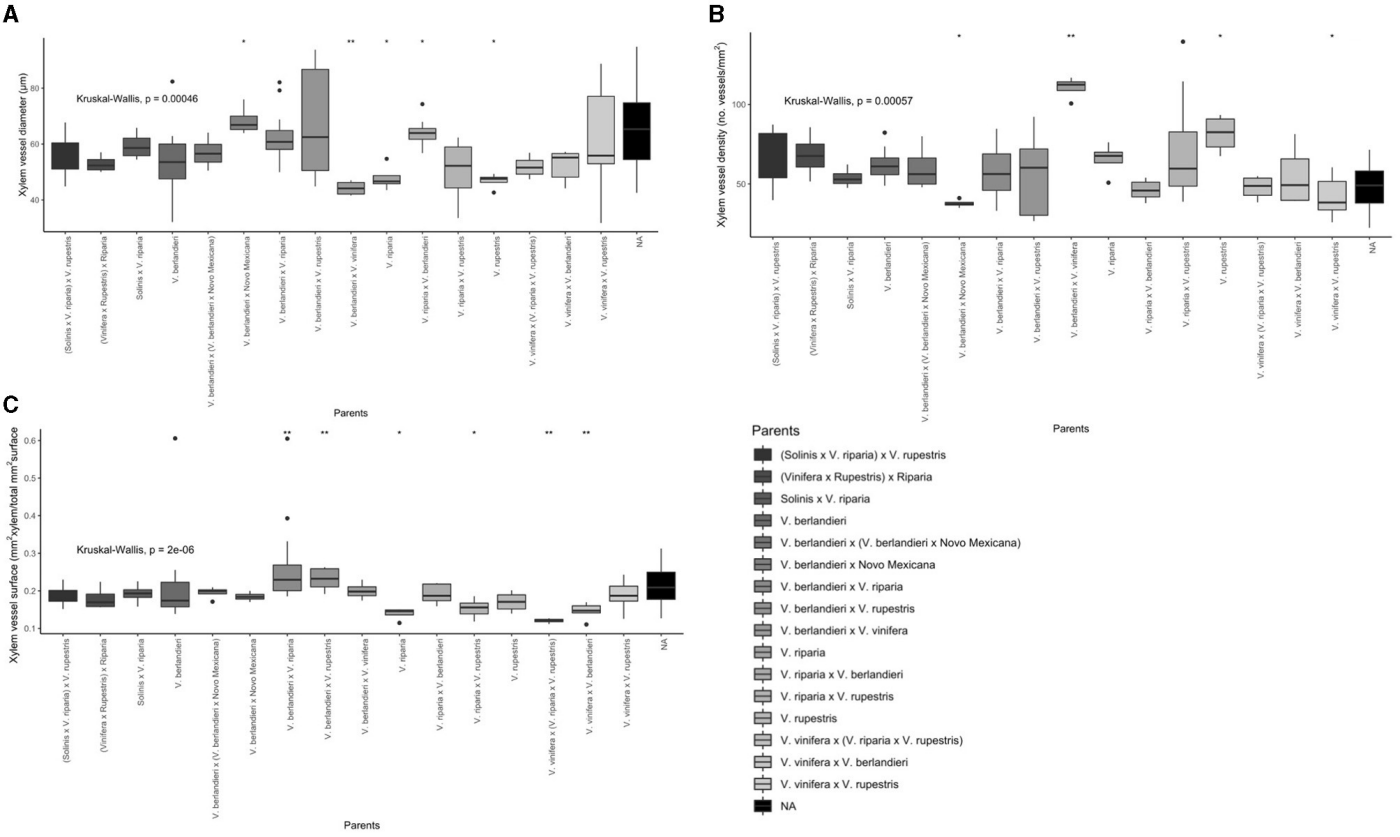
Box plots of the histological traits for each rootstock parent cross (Solinis = *V. longii*). **(A)** Xylem vessel diameter (μm). **(B)** Xylem vessel density (number of vessels/mm^2^ xylem). **(C)** Proportion of stem surface area covered by xylem vessels (mm^2^ xylem vessels/total mm^2^ vascular surface area). Significance (*P < 0.05, **P < 0.01) determined by the Wilcoxon rank sum test.

When comparing rootstock parent crosses rather than individual varieties, all the histological traits were statistically significant. *Vitis berlandieri* × *V. vinifera* showed a significantly narrower vessel diameter (*P* < 0.01) and significantly higher vessel density (*P* < 0.01), but did not statistically differ for vessel surface (Figures 5A–C, Supplementary Table 3, Supplementary Figure 3). *Vitis berlandieri* × Novo Mexicana, *V. riparia, V. riparia* × *V. berlandieri*, and *V. rupestris* also showed statistically different vessel diameters (*P* < 0.05; Figures 5A–C, Supplementary Table 3, Supplementary Figure 3). Of these, *V. berlandieri* × Novo Mexicana and *V. rupestris* had significantly lower vessel density, along with *V. vinifera* × *V. rupestris* (*P* < 0.05; Figures 5A–C, Supplementary Table 3, Supplementary Figure 3). *Vitis riparia, V. riparia* × *V. rupestris, V. berlandieri* × *V. riparia, V. berlandieri* × *V. rupestris, V. vinifera* × (*V. riparia* × *V. rupestris)* and *V. vinifera* × *V. berlandieri* all had a statistically different vessel surface (*P* < 0.05; Figure 5C, Supplementary Table 3).

### Relationship Between Pathogen Tolerance and Xylem Anatomy

**Figure 6:**
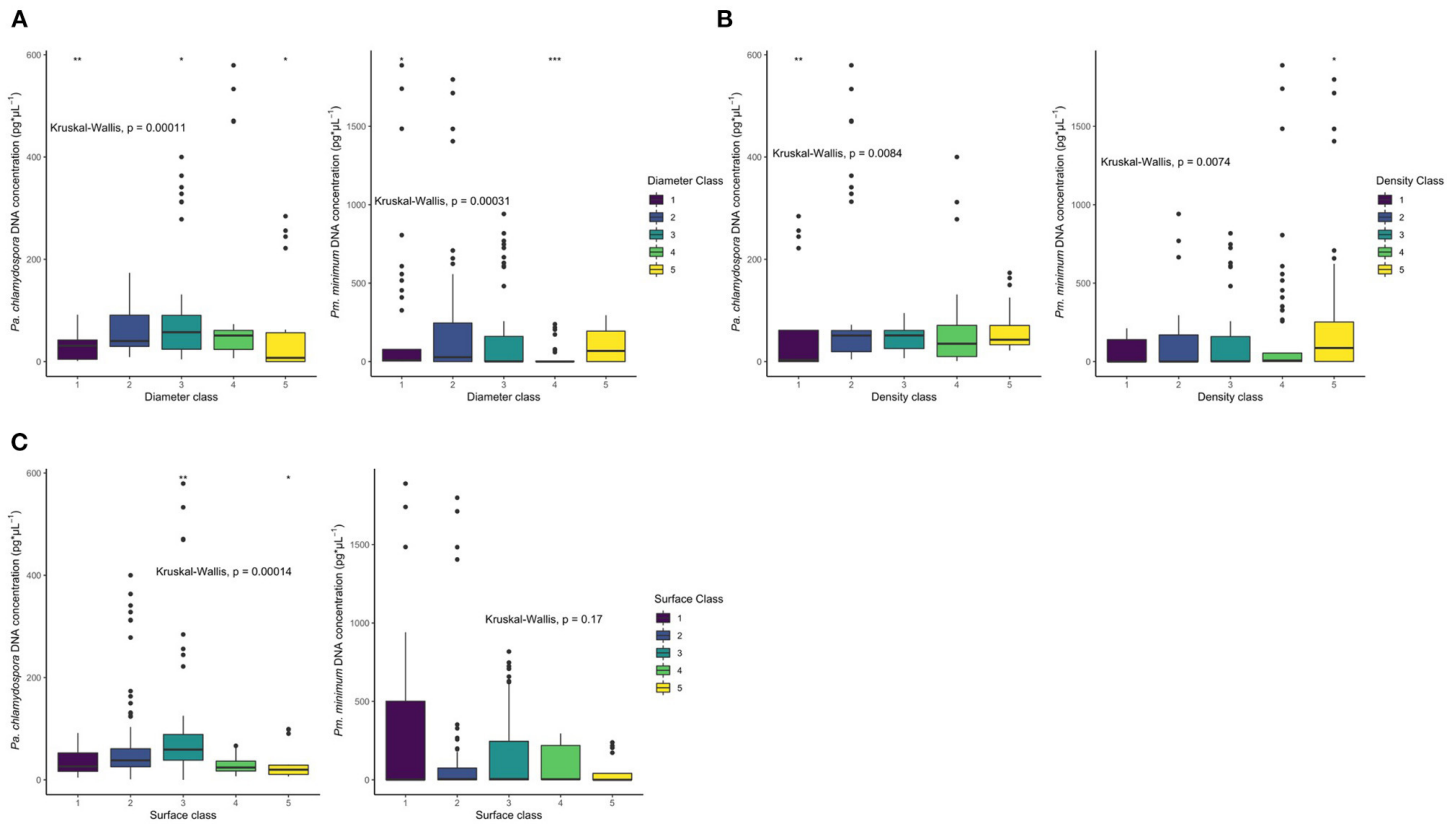
Box plots of the fungal DNA concentration for each histological group. **(A)** The fungal DNA concentration in pg/μl for each diameter class (<44, 45–54, 55–64, 65–74, and 75–84 μm, respectively). Left: *Pa. chlamydospora*. Right: *Pm. minimum*. Color-coded by diameter class. **(B)** The fungal DNA concentration in pg/μl for each density class [ <36, 37–46, 47–56, 57–66, 67, and above (vessels/*mm*^2^), respectively]. Left: *Pa. chlamydospore*. Right: *Pm. minimum*. Color-coded by density class. **(C)** The fungal DNA concentration in pg/μl for each surface class [0.10–0.14, 0.15–0.19, 0.20–24, 0.24–0.30, 0.31 and above (xylem/total *mm*^2^), respectively]. Left: *Pa. chlamydospora*. Right: *Pm. minimum*. Color-coded by Surface class. Surface classes are non-significant for *Pm. minimum* (*P* < 0.05). Significance (*P < 0.05, **P < 0.01, ***P < 0.001) determined by the Wilcoxon rank sum test.

The effect of each xylem parameter classes was strong on *Pa. chlamydospora* DNA concentration (*P* < 0.01; Figures 6A–C, Supplementary Figure 2). A marked effect of xylem vessel diameter and xylem density classes was also observed on *Pm. minimum* DNA concentration, but xylem vessel surface classes had no marked effect (*P* < 0.01; Figures 6A–C). A significantly higher *Pa. chlamydospora* DNA concentration was related to the rootstocks with xylem vessel diameters from 45 to 74 μm. The *Pa. chlamydospora* DNA concentration was also significantly higher for the rootstocks with xylem density values above 37 vessels/mm^2^, and xylem surface values falling within the range of 0.2–0.24 mm^2^ xylem/mm^2^ total surface area (Figures 6A–C, Supplementary Tables 4–6). For *Pm. minimum*, the DNA concentration was significantly higher for the rootstocks with a vessel diameter above 75 μm and the xylem densities above 60 vessels/mm^2^ (Figures 6A–C, Supplementary Tables 7, 8).

## Discussion

This study evaluated the relationship between the xylem anatomy of grapevine rootstocks and their susceptibility to *Pa. chlamydospora* and *Pm. minimum*. This is the first time that this interaction has been studied for *Pm. minimum*. Our results demonstrate the existence of a relationship between xylem anatomy and the ability of both *Pa. chlamydospora* and *Pm. minimum* to differentially colonize the vascular system of a wide range of grapevine rootstocks. We found significant differences between rootstocks for the DNA concentration of both pathogens detected in the inoculated shoots and for the estimated xylem parameters. A significant relation was observed between xylem vessel diameter and density, and the *Pa. chlamydospora* and *Pm. minimum* DNA concentrations. These results also agree with previous research that has studied the xylem vessel diameter effect on compartmentalization efficiency and thus, vascular pathogen movement, using the *V. vinifera*-*Pa. chlamydospora* interaction as a model system (Pouzoulet et al., 2017).

In our study, the susceptibility characterization of grapevine rootstocks was performed by assessing pathogen colonization based on traditional isolation methods in culture medium and DNA quantification by qPCR. The results showed significant differences for the disease incidence between rootstocks by following quantitative methods to estimate the amount of pathogen DNA while no significant differences were observed based on pathogen isolation. Colonization assessment can be hindered by techniques that rely on pathogen isolation from culturing tissue sections on nutritive media. In this context, only the accurate quantification of pathogen biomass can be useful to gain a better understanding of the disease reaction of host genotypes (Gramaje et al., 2013). In a previous study about grapevine tolerance to *Pa. chlamydospora*, fungal colonization was evaluated based on both wood necrotic lesion length and qPCR on inoculated plants (Pouzoulet et al., 2017). Similar analyses have been performed for other perennial plants, such as olive, where the amount of pathogen DNA quantified in different genotypes correlated with susceptibility to vascular pathogen *Verticillium dahliae* (Mercado-Blanco et al., 2003).

Our results showed a significant effect of the xylem vessel diameter of the analyzed rootstocks on *Pa. chlamydospora* and *Pm. minimum* DNA concentrations. Significantly higher *Pa. chlamydospora* concentrations were detected for the diameter range from 55 to 64 μm and surface within the range of 0.2–0.24 mm^2^ xylem vessels/mm^2^. The *Pm. minimum* DNA concentration was significantly higher for a xylem density of above 66 vessels/mm^2^. Some rootstocks like Berlandieri Resseguier 1 and Castel 6736, in which significantly lower pathogen DNA concentrations were detected, also showed significantly narrower vessel diameters.

Previous studies about the grapevine xylem anatomy on cultivars Merlot, Chardonnay, Cabernet Sauvignon, and Thompson Seedless have reported varying xylem vessel diameters and a positive correlation between the number of vessels with diameters above 120 μm and the *Pa. chlamydospora* DNA concentration (Pouzoulet et al., 2017). In general, less efficient pathogen movement restriction was observed for the cultivars proportionally harboring more vessels with a wide diameter, such as Thompson seedless, than for those like Merlot that proportionally displayed narrower diameter vessels (Pouzoulet et al., 2017). The herein obtained conclusions agree with previous reports that consider Merlot to be relatively resistant and Thompson seedless to be more susceptible to esca (Feliciano et al., 2004; Bruez et al., 2013; Murolo and Romanazzi, 2014).

Similar results were obtained when the same cultivars were assessed for Pierce's disease susceptibility (Deyett et al., 2019). From the herein obtained results, it can be speculated that cultivars with wide xylem vessels can be more prone to Pierce's disease decline caused by vascular pathogen *Xylella fastidiosa* (Deyett et al., 2019). Merlot has also been described as being tolerant to other diseases like Flavescence dorée given its ability to compartmentalize vascular vessels by forming tyloses, and also for its higher proportion of narrow vessels (Jelmini et al., 2020).

The relationship between xylem characteristics and the ability of either *Pa. chlamydospora* or *Pm. minimum* to colonize grapevine rootstocks would be useful to better understand the mechanisms associated with GTDs resistance. As indicated in previous studies, this relationship contributes to the hypothesis that a larger xylem vessel would be harder to obstruct (Pouzoulet et al., 2017, 2020a). One key determinant of plant resistance to vascular infections lies in the ability of the host to successfully compartmentalize invaders at the xylem level. Growing evidence supports the notion that the structural properties of the vascular system impact the vulnerability of the host to vascular pathogens (Solla and Gil, 2002; Venturas et al., 2014; Pouzoulet et al., 2017, 2020a).

Recent studies further demonstrate how the grapevine xylem vessel diameter affects *Pa. chlamydospora* susceptibility (Pouzoulet et al., 2020a). In this context, the experimental xylem vessel diameter data of a single Cabernet Sauvignon genotype inoculated with *Pa. chlamydospora* has been used to calibrate a mechanistic stochastic model of pathogen spread and has evidenced that the efficiency of the compartmentalization process in a given xylem vessel is in accordance with its diameter (Pouzoulet et al., 2020a).

The importance of xylem anatomy to determine disease susceptibility has been previously described for other grapevine pathogens that systemically move through the vascular system, such as *X. fastidiosa* (Chatelet et al., 2011). Sun et al. (2013) hypothesized that vessels with wider diameters that harbor many tyloses might be linked with Pierce's disease symptom severity. The role of xylem anatomy in resistance to other pathogens that colonize the vascular system in perennial plants has also been reported for Dutch elm disease. Previous results have shown that a high proportion of vessels above 100 mm in diameter negatively correlate with host disease resistance (Solla and Gil, 2002; Venturas et al., 2014).

Disease tolerance of grapevine rootstocks to *Pa. chlamydospora* and *Pm. minimum* has been assessed by artificial inoculation based on *in vitro*, greenhouse, and field experiments with variable and contradictory results. For example, rootstock Richter 110 was rated as being susceptible under field and *in vitro* experimental conditions, but as tolerant when evaluated in greenhouse trials (Eskalen et al., 2001; Zanzotto et al., 2008; Gramaje et al., 2010). Rootstock 161-49C has been shown to be susceptible under greenhouse conditions and tolerant in field experiments (Eskalen et al., 2001; Gramaje et al., 2010). These variable results about susceptibility to these pathogens in the genetic grapevine pool could relate to their *Vitis* spp. cultivar pedigree. For example, it has been hypothesized that a variable degree of Pierce's susceptibility in *V. vinifera* could be partially attributed to the anatomical features of the host, which are shaped by its pedigree background (Deyett et al., 2019). In our study, significantly narrower vessel diameters were observed for rootstock parents *V. berlandieri* × *V. vinifera, V. riparia, V. riparia* × *V. rupestris*, and *V. rupestris*.

Anatomical measurements have also been linked with the adaptation of the xylem to dehydration in different grapevine cultivars (Pouzoulet et al., 2020b). Many xylem vessels and a large lumen area have been consistently associated with higher hydraulic conductivity and greater vulnerability to drought-induced cavitation (Pouzoulet et al., 2020b). Moreover, such cultivar conditions are consistent with domestication in a semi-arid habitat where a larger number and bigger size diversity of xylem vessels would be needed to transport water and to meet evaporative demand as opposed to cultivars domesticated in temperate forest regions. Evolution under different environmental conditions and domestication practices would explain such different strategies for water transportation and xylem anatomy traits (Pouzoulet et al., 2020b). It is important to consider the relation between drought and pathogen resistance because pathogens have been implicated as a mortality agent in plants weakened from drought (McDowell et al., 2008; Sala, 2010).

Without standard inoculation methodologies for GTD susceptibility assessments, it is useful to identify characteristics to select materials with putative resistance. In this context, the use of xylem characteristics, such as vessel diameter and vessel density, could provide a screening method to identify candidate grapevine rootstocks for further resistance testing. However, further research will be necessary to identify additional environmental effects like the effect of water availability because it may influence xylem development. Morphological traits of the vascular system, such as vessel diameter, are known to present developmental plasticity that responds to environmental factors during plant growth (Lovisolo and Schubert, 1998; Munitz et al., 2018; Pouzoulet et al., 2020b).

In the context of an integrated disease management program against GTDs, growers planting tolerant grapevine cultivars would significantly increase disease control. By providing pathogen-specific data on rootstock susceptibility, growers will have the choice to select adequate planting material in areas where some pathogens are preponderant. The information herein provided will allow researchers to offer growers planting recommendations in the short term and to provide the building blocks for future long-term breeding programs.

## Data Availability Statement

The datasets and code generated for this study can be found in the following github repository https://github.com/cramsing/Ramsing-et-al.-Xylem-anatomy-.

## Author Contributions

DG, JAr, and MB contributed to conception and design of the study. DG and FC provided plant materials. SM and JAg provided the xylem anatomy data. CR organized the database and performed the analysis. CR and MB wrote the manuscript. All authors contributed to read and revise the manuscript and approved the submitted version.

## Funding

This research was founded by FEDER funding through a State Program of I + D + i oriented to the Challenges of Society (RTA2015-00015-C02-00), supported by The National Institute for Agricultural and Food Research and Technology (INIA). DG was supported by the Ramón y Cajal program, Spanish Government (RyC-2017-23098). This research has been developed as a result of a mobility stay funded by the Erasmus+ – KA1 Erasmus Mundus Joint Master Degrees Programme of the European Commission under the PLANT HEALTH Project.

## Conflict of Interest

The authors declare that the research was conducted in the absence of any commercial or financial relationships that could be construed as a potential conflict of interest.

## Publisher's Note

All claims expressed in this article are solely those of the authors and do not necessarily represent those of their affiliated organizations, or those of the publisher, the editors and the reviewers. Any product that may be evaluated in this article, or claim that may be made by its manufacturer, is not guaranteed or endorsed by the publisher.
