# Supplementary material for: Relationship Between the Xylem Anatomy of Grapevine Rootstocks and Their Susceptibility to Phaeoacremonium minimum and Phaeomoniella chlamydospora
Source: Front Plant Sci. 2021 Oct 12;12:726461. doi: 10.3389/fpls.2021.726461 (PMC8546399; doi:10.3389/fpls.2021.726461)
Supplement: Supplementary file 1 [file Data_Sheet_1.zip › Supplementary Tables and Figures Captures.docx]

Supplementary Material

## Supplementary Tables

**Supplementary Table 1.** Post-hoc Wilcoxon rank sum test results for *Pa. chlamydospora* and *Pm. minimum* DNA concentration between rootstock varieties. (* P <0.05, ** P < 0.01,*** P < 0.001,**** P < 0.0001)

| **Parameter** | **Rootstock** | **Average *Pa. chlamydospora* DNA concentration** | **Standard Error** | **P-value** | **Level** | **Method** |
| --- | --- | --- | --- | --- | --- | --- |
| *Pa. chlamydospora c*oncentration | BERLANDIERI RESSEGUIER 1 | 2.375 | 0.4073798 | 0.00 | **** | Wilcoxon |
|  | CASTEL 196-17 | 94.1875 | 21.4911563 | 0.04 | * | Wilcoxon |
|  | CASTEL 6971 | 196.85462 | 52.9247207 | 0.00 | ** | Wilcoxon |
|  | COUDERC 1202 | 83.85833 | 35.9723409 | 0.04 | * | Wilcoxon |
|  | COUDERC 161-49 | 209.01842 | 65.3637688 | 0.00 | ** | Wilcoxon |
|  | ESCUELA MONTPELLIER 333 | 11.93333 | 1.7252726 | 0.00 | *** | Wilcoxon |
|  | EVEX JEREZ 13-5 | 9.0875 | 0.9419656 | 0.00 | *** | Wilcoxon |
|  | MILLARDET GRASSET 420A | 163.21755 | 34.2978529 | 0.00 | **** | Wilcoxon |
|  | RICHTER 110 | 7.3 | 0.1683251 | 0.02 | * | Wilcoxon |
|  | RUGGERI 140 | 101.27143 | 10.0175492 | 0.00 | ** | Wilcoxon |
|  | TELEKI-KOBER 5BB | 61.525 | 1.9689685 | 0.02 | * | Wilcoxon |
|  | **Rootstock** | **Average *Pm. minimum* DNA concentration** | **Standard Error** | **P-value** | **Level** | **Method** |
| *Pm. minimum* concentration | CASTEL 196-17 | 916.532857 | 325.841709 | 0.0069 | ** | Wilcoxon |
|  | CASTEL 6736 | 0.40620204 | 0.19384987 | 0.041 | * | Wilcoxon |
|  | CASTEL 6971 | 0.02383333 | 0.0153632 | 0.0229 | * | Wilcoxon |
|  | FERCAL | 483.083882 | 78.1669064 | 2.60E-05 | **** | Wilcoxon |
|  | GREZOT G1 | 0.29260583 | 0.12006404 | 0.0282 | * | Wilcoxon |
|  | GRIMALDI 791 | 0.52790909 | 0.28050569 | 0.0038 | ** | Wilcoxon |
|  | RG9 | 249.50525 | 21.6276856 | 0.0204 | * | Wilcoxon |
|  | RIPARIA GRAND GLABRE | 879.239458 | 186.98062 | 3.20E-06 | **** | Wilcoxon |
|  | TELEKI-KOBER 5BB | 0.03648357 | 0.01896448 | 0.0016 | ** | Wilcoxon |

**Supplementary Table 2**. Post-hoc Wilcoxon rank sum test/T-test results for histological traits (diameter, density, and surface) between rootstock varieties. (* P <0.05, ** P < 0.01,*** P < 0.001,**** P < 0.0001)

| **Parameter** | **Rootstock** | **Average diameter** | **Standard Error** | **P-value** | **Level** | **Method** |
| --- | --- | --- | --- | --- | --- | --- |
| Diameter (μm) | BERLANDIERI RESSEGUIER 1 | 44.64751 | 4.651431 | 0.0308 | * | Wilcoxon |
|  | BLANCHARD 1 | 44.23922 | 1.355428 | 0.0067 | ** | Wilcoxon |
|  | CASTEL 6736 | 41.15957 | 3.215151 | 0.0051 | ** | Wilcoxon |
|  | RG4 | 81.68924 | 5.488645 | 0.0046 | ** | Wilcoxon |
|  | RG9 | 75.04985 | 6.820466 | 0.0264 | * | Wilcoxon |
|  | RICHTER 110 | 83.56721 | 7.314207 | 0.0071 | ** | Wilcoxon |
|  | RICHTER 31 | 68.38655 | 2.66566 | 0.0386 | * | Wilcoxon |
|  | RIPARIA GRAND GLABRE | 47.88715 | 2.398767 | 0.0498 | * | Wilcoxon |
|  | RUPESTRIS FORT WORTH 1 | 46.79286 | 1.433323 | 0.0285 | * | Wilcoxon |
|  | **Rootstock** | **Average density** | **Standard Error** | **P-value** | **Level** | **Method** |
| Density (number of vessels/$\mathrm{mm}^{2}$ xylem) | BLANCHARD 1 | 110.54578 | 3.496849 | 3.10E-05 | **** | T-test |
|  | CASTEL 7605 | 47.20843 | 3.064424 | 0.0035 | ** | T-test |
|  | CASTEL 6971 | 44.66171 | 2.559082 | 0.0244 | * | T-test |
|  | COUDERC 1202 | 31.09448 | 3.626921 | 0.0043 | ** | T-test |
|  | COUDERC 161-49 | 47.56743 | 3.44124 | 0.0427 | * | T-test |
|  | ESCUELA MONTPELLIER 333 | 41.20552 | 1.679411 | 6.20E-06 | **** | T-test |
|  | RG4 | 34.08495 | 4.267066 | 0.0051 | ** | T-test |
|  | RG9 | 41.98223 | 4.788859 | 0.0344 | * | T-test |
|  | RICHTER 110 | 35.16926 | 6.717387 | 0.0365 | * | T-test |
|  | RICHTER 31 | 37.71413 | 1.26495 | 8.70E-10 | **** | T-test |
|  | RUGGERI 140 | 76.36121 | 5.912059 | 0.0468 | * | T-test |
|  | RUPESTRIS FORT WORTH 1 | 81.48071 | 6.1202 | 0.0255 | * | T-test |
|  | **Rootstock** | **Average surface** | **Standard Error** | **P-value** | **Level** | **Method** |
| Surface ($\mathrm{mm}^{2}$ xylem vessels/total $\mathrm{mm}^{2}$ vascular surface area | CASTEL 6736 | 0.1422682 | 0.01020782 | 0.0184 | * | Wilcoxon |
|  | ESCUELA MONTPELLIER 333 | 0.1388835 | 0.01025815 | 0.0143 | * | Wilcoxon |
|  | GRIMALDI 791 | 0.1203612 | 0.00318316 | 0.0021 | ** | Wilcoxon |
|  | RG4 | 0.2551508 | 0.02716858 | 0.0296 | * | Wilcoxon |
|  | RG9 | 0.2591055 | 0.0115393 | 0.0074 | ** | Wilcoxon |
|  | RICHTER 110 | 0.2464315 | 0.00947623 | 0.0131 | * | Wilcoxon |
|  | RIPARIA GRAND GLABRE | 0.1391595 | 0.00837118 | 0.0143 | * | Wilcoxon |
|  | RUGGERI 267 | 0.2592689 | 0.02472138 | 0.0137 | * | Wilcoxon |
|  | SO4 | 0.3567061 | 0.09305074 | 0.0199 | * | Wilcoxon |

**Supplementary Table 3.** Post-hoc Wilcoxon rank sum test for histological traits (diameter, density, and surface) between rootstock parent crosses. (* P <0.05, ** P < 0.01,*** P < 0.001,**** P < 0.0001)

| **Parameter** | **Cross** | **P-value** | **Level** | **Method** |
| --- | --- | --- | --- | --- |
| Diameter | V. berlandieri x Novo Mexicana | 0.0386121200388599 | * | Wilcoxon |
|  | V. berlandieri x V. vinifera | 0.00674065406362805 | ** | Wilcoxon |
|  | V. riparia | 0.0498268385111804 | * | Wilcoxon |
|  | V. riparia x V. berlandieri | 0.0410701036485307 | * | Wilcoxon |
|  | V. rupestris | 0.0284944942847973 | * | Wilcoxon |
| Density | **Cross** | **P-value** | **Level** | **Method** |
|  | V. berlandieri x Novo Mexicana | 0.0155472869516844 | * | Wilcoxon |
|  | V. berlandieri x V. vinifera | 0.00162126379264457 | ** | Wilcoxon |
|  | V. rupestris | 0.0162119449372604 | * | Wilcoxon |
|  | V. vinifera x V. rupestris | 0.0355635188250109 | * | Wilcoxon |
| Surface | **Cross** | **P-value** | **Level** | **Method** |
|  | V. berlandieri x V. riparia | 0.00194554559928968 | ** | Wilcoxon |
|  | V. berlandieri x V. rupestris | 0.00608403637158635 | ** | Wilcoxon |
|  | V. riparia | 0.0142895336068948 | * | Wilcoxon |
|  | V. riparia x V. rupestris | 0.0110350035688186 | * | Wilcoxon |
|  | V. vinifera x (V. riparia x V. rupestris) | 0.00209985847587919 | ** | Wilcoxon |
|  | V. vinifera x V. berlandieri | 0.00334371339721088 | ** | Wilcoxon |

**Supplementary Table 4**. The average *Pa. chlamydospora* DNA concentration (pg/μl) for each diameter class and their Kruskal-Wallis groups

| **Parameter** | **Class** | **Average *Pa. chlamydospora* DNA concentration (pg/μl)** | **Significance Group (Kruskal-Wallis)** |
| --- | --- | --- | --- |
| Diameter | 1 (>44μm) | 87.71 ± 3.48 | b |
|  | 2 (45-54μm) | 134.00 ± 6.65 | a |
|  | 3 (55-64μm) | 135.96 ± 11.43 | a |
|  | 4 (65-74μm) | 121.01 ± 18.30 | a |
|  | 5 (<75μm) | 82.73 ± 22.28 | b |

**Supplementary Table 5**. The average *Pm. minimum* DNA concentration (pg/μl) for each diameter class and their Kruskal-Wallis groups

| **Parameter** | **Class** | **Average *Pm. minimum* DNA concentration (pg/μl)** | **Significance Group (Kruskal-Wallis)** |
| --- | --- | --- | --- |
| Diameter | 1 (<44μm) | 140.14 ± 71.62 | a |
|  | 2 (45-54μm) | 125.79 ± 55.85 | ab |
|  | 3 (55-64μm) | 114.63 ± 32.13 | b |
|  | 4 (65-74μm) | 78.71 ± 9.81 | c |
|  | 5 (<75μm) | 109.88 ± 22.00 | bc |

**Supplementary Table 6**. The average *Pa. chlamydospora* DNA concentration (pg/μl) for each density class and their Kruskal-Wallis groups

| **Parameter** | **Class** | **Average *Pa. chlamydospora* DNA concentration (pg/μl)** | **Significance Group (Kruskal-Wallis)** |
| --- | --- | --- | --- |
| Density | 1 (>36) | 66.03 ± 28.02 | c |
|  | 2 (37-46) | 123.23 ± 24.39 | ab |
|  | 3 (47-56) | 120.59 ± 3.38 | ab |
|  | 4 (57-66) | 110.37 ± 8.22 | b |
|  | 5 (<67) | 135.67 ± 6.20 | a |

**Supplementary Table 7**. The average *Pm. minimum* DNA concentration (pg/μl) for each density class and their Kruskal-Wallis groups

| **Parameter** | **Class** | **Average *Pm. minimum* DNA concentration (pg/μl)** | **Significance Group (Kruskal-Wallis)** |
| --- | --- | --- | --- |
| Density | 1 (>36) | 83.67 ± 24.46 | b |
|  | 2 (37-46) | 103.90 ± 34.43 | b |
|  | 3 (47-56) | 104.45 ± 21.65 | b |
|  | 4 (57-66) | 126.47 ± 52.40 | a |
|  | 5 (<67) | 140.97 ± 80.71 | a |

**Supplementary Table 8**. The average *Pa. chlamydospora* DNA concentration (pg/μl) for each surface class and their Kruskal-Wallis groups

| **Parameter** | **Class** | **Average *Pa. chlamydospora* DNA concentration (pg/μl)** | **Significance Group (Kruskal-Wallis)** |
| --- | --- | --- | --- |
| Surface | 1 (.10-.14) | 96.70 ± 4.74 | bc |
|  | 2 (.15-.19) | 118.56 ± 7.85 | b |
|  | 3 (.20-.24) | 145.77 ± 16.64 | a |
|  | 4 (.25-.30) | 84.78 ± 5.11 | bc |
|  | 5 (<.31) | 82.13 ± 7.56 | c |

## Supplementary Figures

**
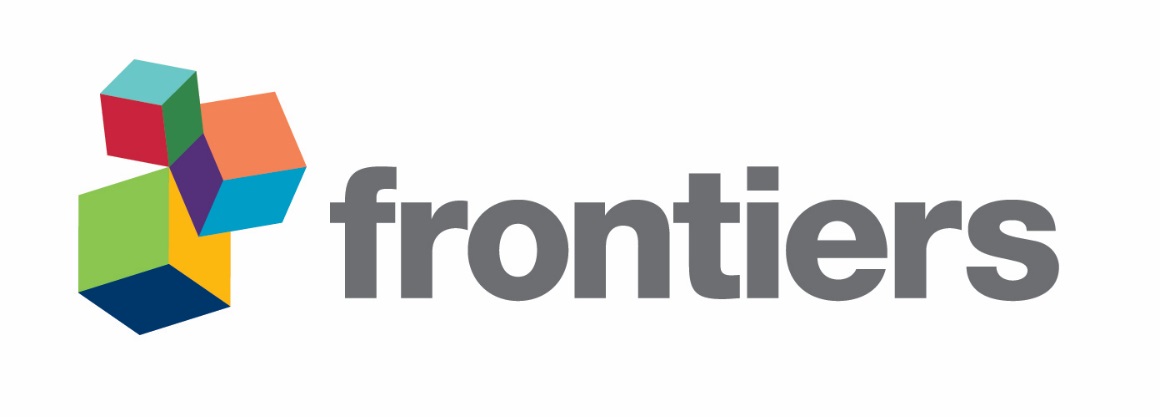
**

**Supplementary Figure 1.** Dot plots of *Pa. chlamydospora* and *Pm. minimum* percent incidence (number of infected samples/number total analyzed samples) for each rootstock variety. A) *Pa. chlamydospora* incidence B) *Pm. minimum* percent incidence

**Supplementary Figure 2**. Box plots of fungal DNA concentration (pg/μL) found in differing grape rootstock varieties equivalent to Figure 1, but color coded by A) density class and B) surface class.

**Supplementary Figure 3**. Histological trait comparison between rootstock parent cross and rootstock variety. A) Xylem vessel diameter plotted by rootstock parent cross (left) and rootstock variety (right). Color coded by rootstock parent cross. B) Xylem vessel density plotted by rootstock parent cross (left) and rootstock variety (right). Color coded by rootstock parent cross. C) Xylem vessel surface plotted by rootstock parent cross (left) and rootstock variety (right). Color coded by rootstock parent cross. Significance (*) determined by Wilcoxon rank sum test (P<0.05).
