# Supplementary figures and images for: Relationship Between the Xylem Anatomy of Grapevine Rootstocks and Their Susceptibility to Phaeoacremonium minimum and Phaeomoniella chlamydospora
Source: Front Plant Sci. 2021 Oct 12;12:726461. doi: 10.3389/fpls.2021.726461 (PMC8546399; doi:10.3389/fpls.2021.726461)

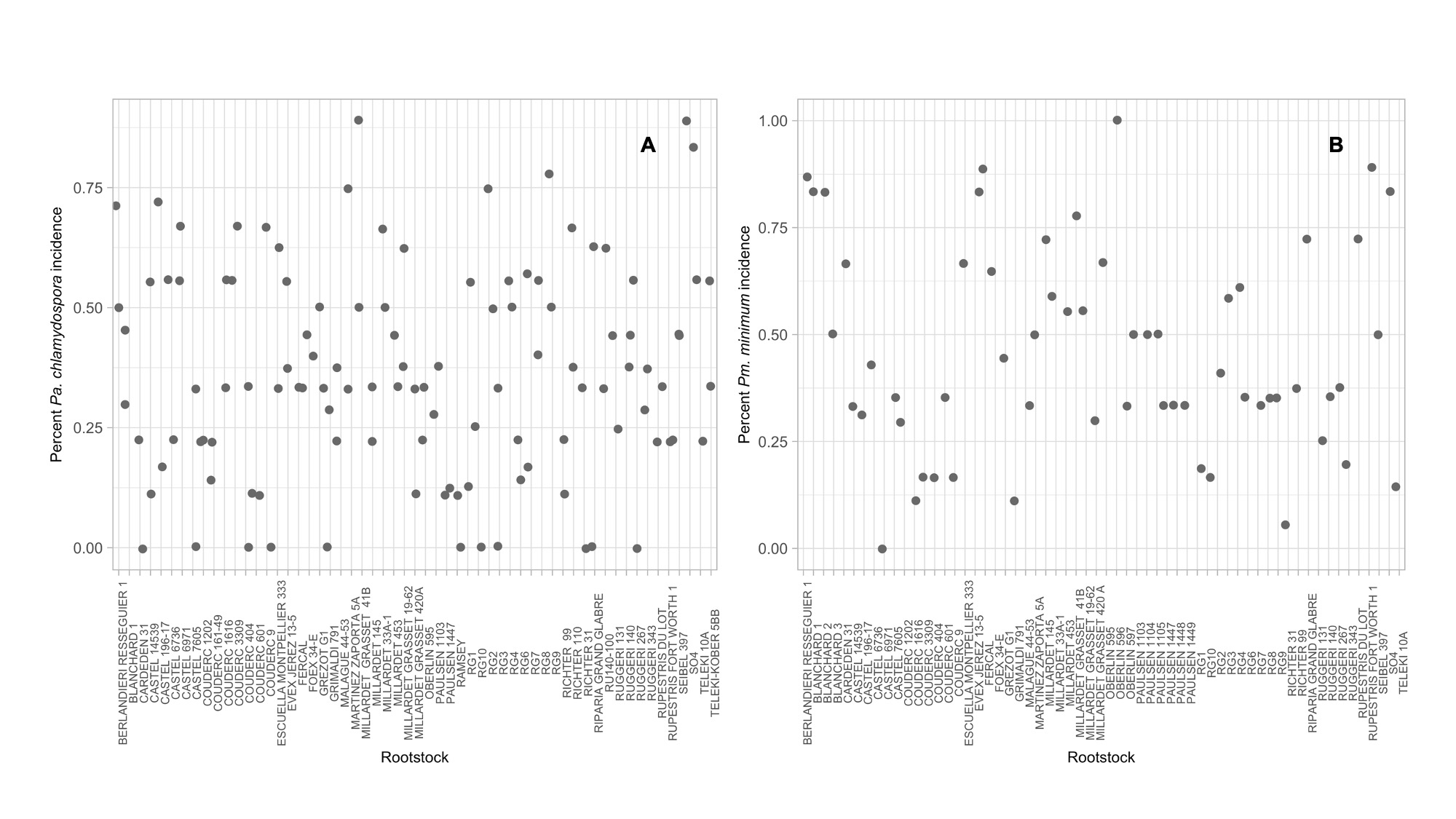

Supplement: Supplementary file 1 [file Data_Sheet_1.zip › Supplementary Figure 1.JPEG]

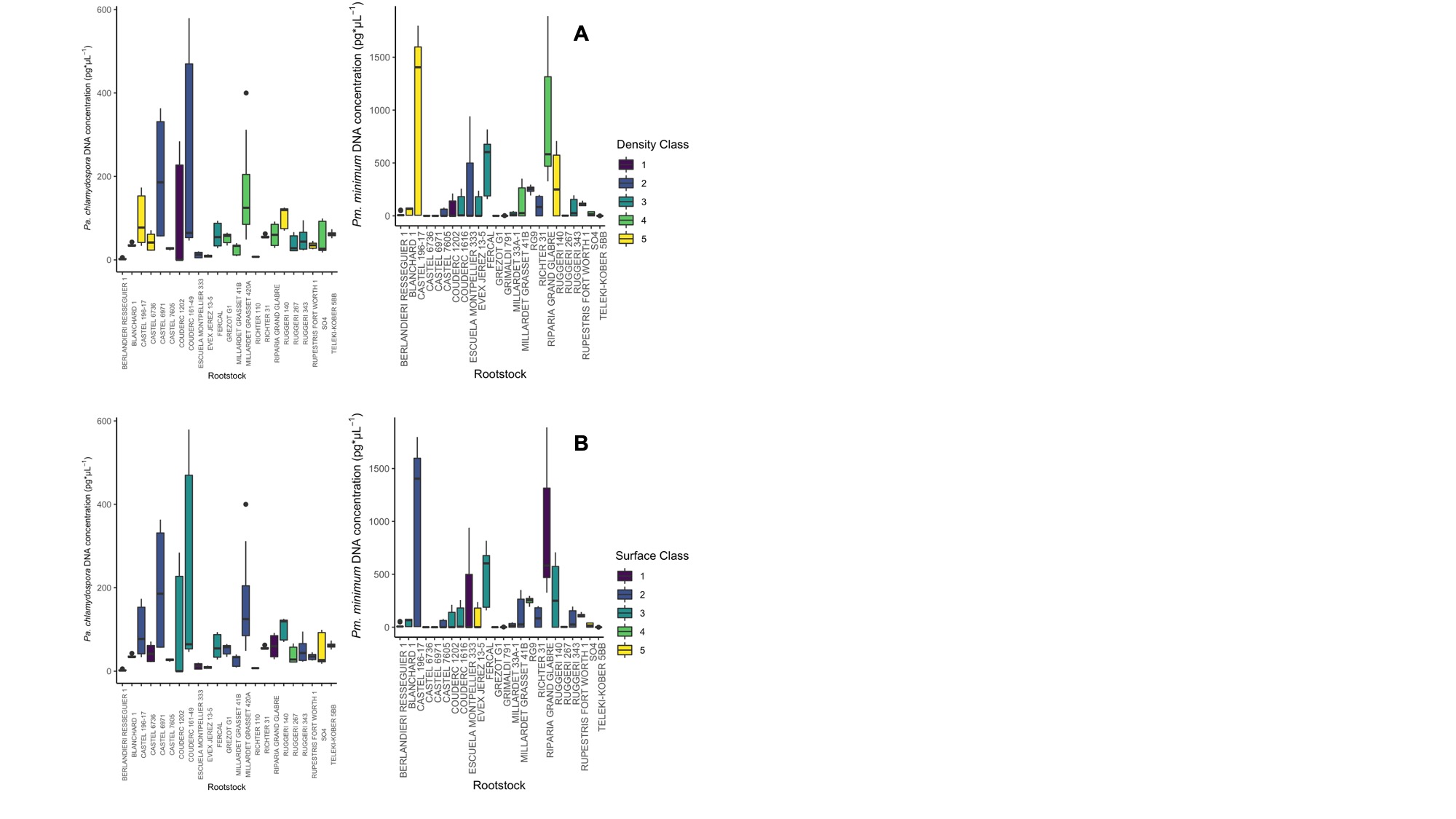

Supplement: Supplementary file 1 [file Data_Sheet_1.zip › Supplementary Figure 2.JPEG]

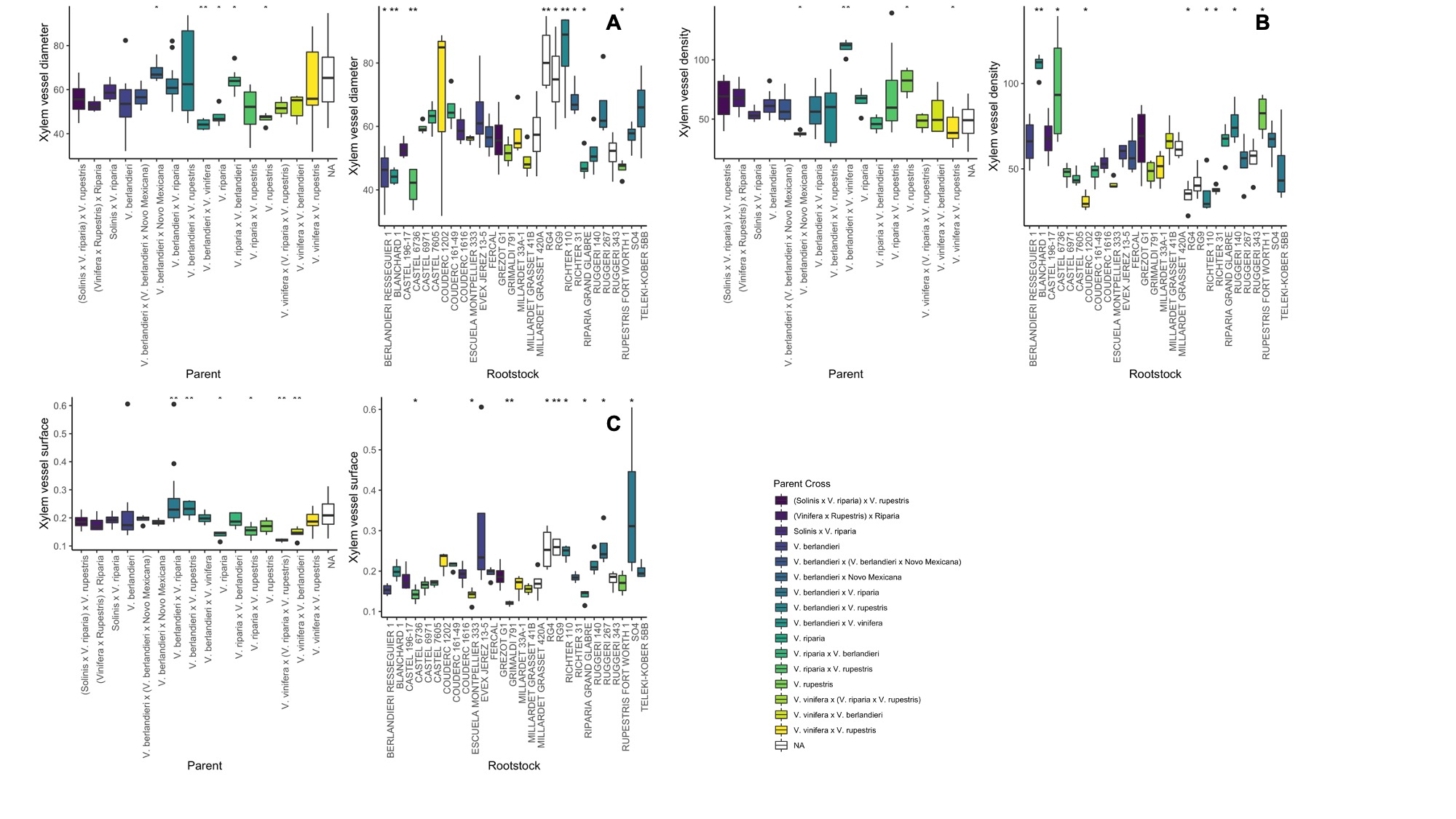

Supplement: Supplementary file 1 [file Data_Sheet_1.zip › Supplementary Figure 3.JPEG]
